# Praeruptorin B Mitigates the Metastatic Ability of Human Renal Carcinoma Cells through Targeting CTSC and CTSV Expression

**DOI:** 10.3390/ijms21082919

**Published:** 2020-04-22

**Authors:** Chia-Liang Lin, Tung-Wei Hung, Tsung-Ho Ying, Chi-Jui Lin, Yi-Hsien Hsieh, Chien-Min Chen

**Affiliations:** 1 Institute of Biochemistry, Microbiology and Immunology, Chung Shan Medical University, Taichung 40201, Taiwan; hiking003@hotmail.com (C.-L.L.); c30205@cgmh.org.tw (C.-J.L.); 2 Department of Medicine, Mackay Medical College, New Taipei City 252, Taiwan; 3 Division of Nephrology, Department of Medicine, Chung Shan Medical University Hospital, Taichung 40201, Taiwan; a6152000@ms34.hinet.net; 4 School of Medicine, Chung Shan Medical University, Taichung 40201, Taiwan; 5 Department of Obstetrics and Gynecology, Chung Shan Medical University Hospital, Taichung 40201, Taiwan; ying.steve@gmail.com; 6 Department of Obstetrics and Gynecology, School of Medicine, College of Medicine, Chung Shan Medical University, Taichung 40201, Taiwan; 7 Institute of Medicine, Chung Shan Medical University, Taichung 40201, Taiwan; 8 Clinical Laboratory, Chung Shan Medical University Hospital, Taichung 40201, Taiwan; 9 Division of Neurosurgery, Department of Surgery, Changhua Christian Hospital, Changhua 50006, Taiwan; 10 School of Medicine, Kaohsiung Medical University, Kaohsiung 80708, Taiwan; 11 College of Nursing and Health Sciences, Dayeh University, Changhua 51591, Taiwan

**Keywords:** renal cell carcinoma, Praeruptorin-B, migration, invasion, CTSC, CTSV

## Abstract

Renal cell carcinoma (RCC) is the most common adult kidney cancer, and accounts for 85% of all cases of kidney cancers worldwide. Praeruptorin B (Pra-B) is a bioactive constituent of *Peucedanum praeruptorum* Dunn and exhibits several pharmacological activities, including potent antitumor effects. However, the anti-RCC effects of Pra-B and their underlying mechanisms are unclear; therefore, we explored the effects of Pra-B on RCC cells in this study. We found that Pra-B nonsignificantly influenced the cell viability of human RCC cell lines 786-O and ACHN at a dose of less than 30 μM for 24 h treatment. Further study revealed that Pra-B potently inhibited the migration and invasion of 786-O and ACHN cells, as well as downregulated the mRNA and protein expression of cathepsin C (CTSC) and cathepsin V (CTSV) of 786-O and ACHN cells. Mechanistically, Pra-B also reduced the protein levels of phospho (p)-epidermal growth factor receptor (EGFR), p-mitogen-activated protein kinase kinase (MEK), and p-extracellular signal-regulated kinases (ERK) in RCC cells. In addition, Pra-B treatment inhibited the effect of EGF on the upregulation of EGFR–MEK–ERK, CTSC and CTSV expression, cellular migration, and invasion of 786-O cells. Our findings are the first to demonstrate that Pra-B can reduce the migration and invasion ability of human RCC cells through suppressing the EGFR-MEK-ERK signaling pathway and subsequently downregulating CTSC and CTSV. This evidence suggests that Pra-B can be developed as an effective antimetastatic agent for the treatment of RCC.

## 1. Introduction

Kidney cancer accounts for approximately 3% of adult malignancies, and yearly kidney cancer incidence rates are increasing in more developed regions of the world [1]. To date, most patients with kidney cancer have an excellent prognosis in the early stage; however, prior research has noted limited survival and poor outcomes in patients, which has been attributed to advanced or distant tumor metastasis [2]. At present, surgical intervention combined with chemotherapy is the standard therapy for patients with tumor metastasis; nonetheless, adverse side effects and drug resistance remain obstacles [3]. Therefore, the development of antimetastatic reagents for renal cell carcinoma (RCC) potentially improves current RCC treatment strategies.

*Peucedanum praeruptorum* DUNN (*P. praeruptorum*), a traditional Chinese medical herb, is well known for its pharmacological function in treating headaches, coughing, and vomiting. To date, many studies have indicated that angular-type pyranocoumarins and furanocoumarins are major constituents of dried roots of *P. praeruptorum* [4], and pharmacological studies have shown that these compounds may possess a wide variety of activities, such as anti-inflammatory [5], antiasthma [6], and neuroprotective [7]. Praeruptorins are major bioactive members of pyranocoumarin and can be divided into five species: A, B, C, D, and E. Praeruptorin A (Pra-A) is reported to exert a protective effect on osteoporosis through inhibiting the p38/AKT/c-Fos/NAFTc1 pathway [8]. Pra-C was observed to mitigate cardiac damage and have a clear effect on blood pressure in spontaneously hypertensive rats, suggesting its potential as a novel drug for the treatment and prevention of cardiovascular diseases [9]. One study reported that Pra-B inhibits sterol regulatory element-binding proteins (SREBPs) to improve hyperlipidemia and insulin resistance [10]. Moreover, Pra-A and Pra-C were indicated to possess cytotoxic activity and induce apoptosis against lymphocytic leukemia cells [7,11]. Another study demonstrated that praeruptorins enhanced the sensitivity of doxorubicin, paclitaxel, and vincristine in cancer cells [12], suggesting a potential anticancer effect. However, the effects and molecular mechanisms of the antitumor effect of Pra-B on RCC have thus far not been clarified.

The extracellular matrix (ECM) is a highly dynamic and continuous process during composition, reorganization, and degradation. It has the function of maintaining tissue homeostasis and is responsible for cell–cell interaction, cell migration, and cell proliferation. However, the dysregulation of ECM’s dynamics process may lead to the development of different diseases [13]. ECM degradation by extracellular proteinases is a key step in tumor cell invasion and metastasis. Among them, the expression of matrix metalloproteinase (MMP) activity has been highly correlated with cancer cell metastasis and has thus been considered a target for anticancer drugs in the literature [14,15]. Cysteine cathepsins are proteases that are frequently secreted into the extracellular environment and during the activation of MMPs, which regulate the invasion of cancer cells [16]. Studies have implicated that overexpression of CTSC and CTSV expression in various different malignant tumors, such as breast ductal carcinoma, colorectal carcinomas, and pancreatic [17,18,19], and it was suggested to be associated with poor prognosis in HCC [20]. Moreover, Zhang et al. observed that CTSC mediated hepatoma tumor cell proliferation and metastasis by activation of the TNF-α/p38 MAPK pathway [21]. Keegan et al. demonstrated that TNF-α induced monocyte-endothelial cell and increased the CTSV activity through dependency on JNK signaling pathways in cardiovascular disease [22]. Although these studies have discovered CTSV and CTSC involved in tumor progression, the intracellular signaling cascades linking the Pra-B regulate the levels of CTSV and CTSC in RCC cells for further investigation.

In this study, we investigate the inhibitory effect of Pra-B on migration and invasion in RCC and further identify underlying molecular mechanisms for these effects. Our results demonstrated that Pra-B suppressed cellular motility through reducing the mRNA and protein expression of CTSC/CTSV and suppressing the EGFR–MEK–ERK signaling pathway. This suggested that Pra-B has potential as an antimetastatic agent in human RCC cells.

## 2. Results

### 2.1. Effect of Pra-B on Cell Viability and Cytotoxicity in Human RCC Cells and Normal HK2 Cells

**Figure 1:**
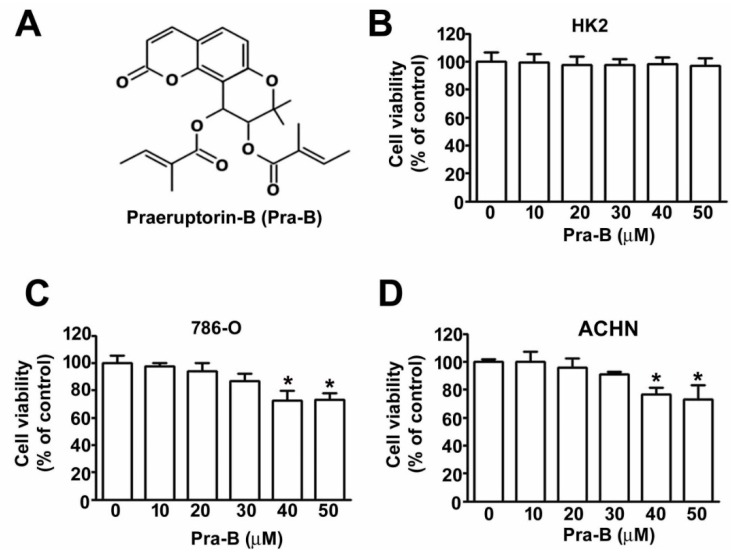
Effects of Praeruptorin B (Pra-B) on cell viability of human renal cell carcinoma (RCC) cells and normal HK2 cells. (**A**) Structure of praeruptorin B (Pra-B); (**B**–**D**) cell viability of proximal tubule normal HK2 cells and RCC cell lines 786-O and ACHN treated with various concentrations of Pra-B (0, 10, 20, 30, 40, and 50 μM) for 24 h. * *p* < 0.05 relative to the control. Data are presented in terms of mean ± SD, as determined in at least three independent experiments.

Figure 1A illustrates the chemical structure of Pra-B. An MTT assay was used to examine the cell viability and cytotoxicity of various concentrations of Pra-B (0, 10, 20, 30, 40, and 50 μM) for 24 h, which led to the observation that treated with Pra-B doses higher than 40 μM, resulted in the reduction of cell viability in 786-O and ACHN cells, but doses lower than 30 μM did not induce cytotoxicity (Figure 1C,D). However, Pra-B exhibited no cytotoxicity in human HK-2 cells (Figure 1B). These results revealed that Pra-B is not toxic to human RCC cells at 30 μM; therefore, serial Pra-B concentrations in the range of 0–30 μM were used in subsequent experiments.

### 2.2. Pra-B Inhibited Cell Migration and Invasion in 786-O and ACHN Cells

**Figure 2:**
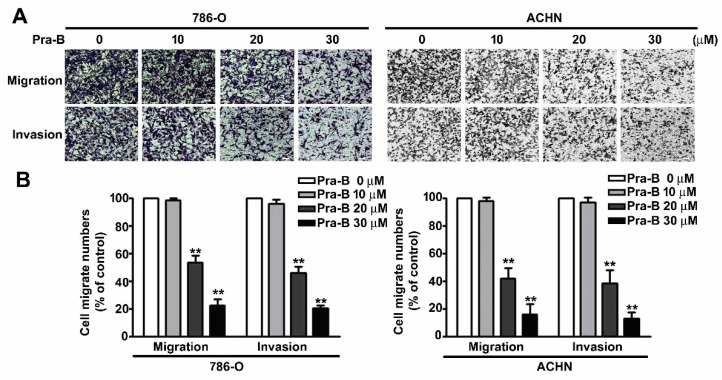
Inhibitory effects of Pra-B on cell migration and invasion of 786-0 and ACHN cell. (**A**) Cells were treated with various concentrations of Pra-B (0, 10, 20, and 30 μM) for 24 h followed by the analysis of 786-0 and ACHN cell migration and invasion by using an in vitro migration and Matrigel-based invasion assay. (**B**) Quantification of migrating cells presented in terms of percentage of control (0 μM) is shown as a histogram, as determined in at least three independent experiments. ** *p* < 0.01 relative to the control. Scale bar = 50 μm.

To examine the effect of Pra-B on cellular migration and invasion ability in renal cell carcinoma, we treated 786-O and ACHN cells with various concentrations of Pra-B (0–30 μM) for 24 h. Our results revealed that an increasing dose of Pra-B significantly reduced 786-O and ACHN cell migration and invasion ability (Figure 2A). On the basis of quantitative assessment, the migrate cell numbers of cells treated with 20 and 30 μM of Pra-B was reduced by 42% and 79% in 786-O cells, and 60% and 82% in ACHN cells, respectively. In addition, such doses of Pra-B inhibited 58% and 80% of cell invasion in 786-O cells, and 62% and 86% in ACHN cells, respectively (Figure 2B). These results demonstrated that Pra-B has an inhibitory effect on 786-O and ACHN cell migration and invasion capacities.

### 2.3. Pra-B Inhibits the Expression of CTSC and CTSV in 786-O Cells

**Figure 3:**
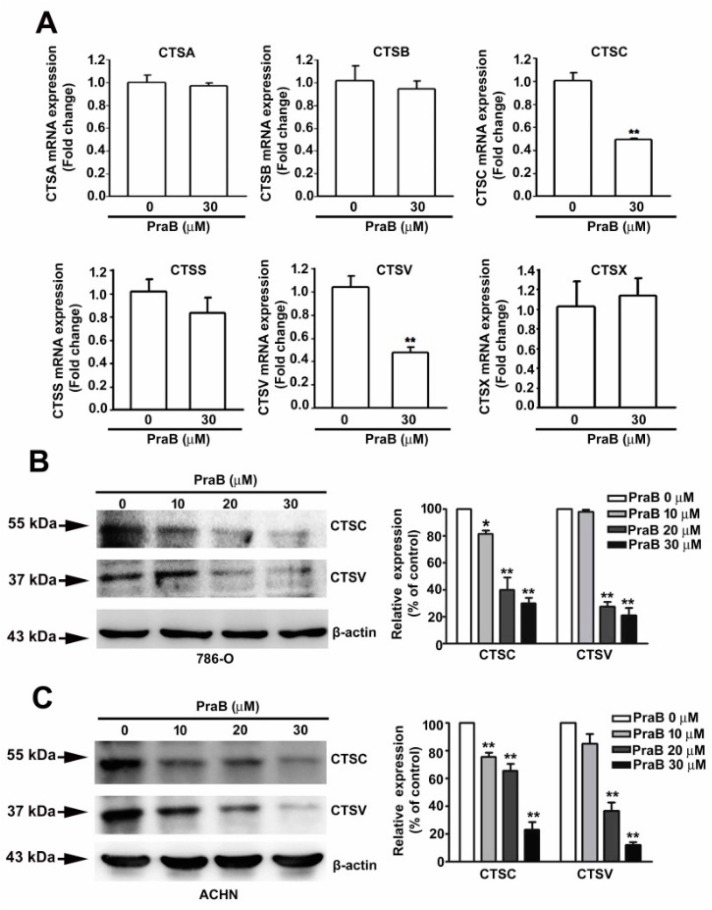
Inhibitory effects of Pra-B on cathepsin C (CTSC) and cathepsin V (CTSV) expression in RCC cells. (**A**) 786-O cells were treated with Pra-B (0 and 30 μM) for 24 h, after which the cells were harvested for the detection of CTS mRNA levels through quantitative real-time PCR. (**B**,**C**) 786-O and ACHN cells were treated with various concentrations of Pra-B (0, 10, 20, and 30 μM) for 24 h, and protein levels were subsequently analyzed using immunoblotting. The histogram represents the densitometric analysis of CTSC and CTSV protein expression. β-actin was used as the loading control. * *p* < 0.05, ** *p* < 0.01 relative to the control. Data are presented in terms of mean ± SD, as determined in at least three independent experiments.

Cathepsin-related proteins have been broadly implicated in cancer [23], and we even previously reported CTSC as being a target in the suppression of RCC migration and invasion [24]. To identify the effect of Pra-B on CTS-related mRNA expression, we treated 786-O cells with Pra-B (30 μM) for 24 h and used qRT-PCR analysis to screen the expression of CTS-related gene expression. The results indicated that the mRNA expression of CTSC and CTSV was significantly reduced in response to Pra-B treatment (Figure 3A). Furthermore, Western blot analysis also demonstrated that the protein expression of CTSC and CTSV was reduced upon Pra-B treatment in 786-O and ACHN cells (Figure 3B,C).

### 2.4. Pra-B Suppressed Activation of the MEK–ERK Signaling Pathway

**Figure 4:**
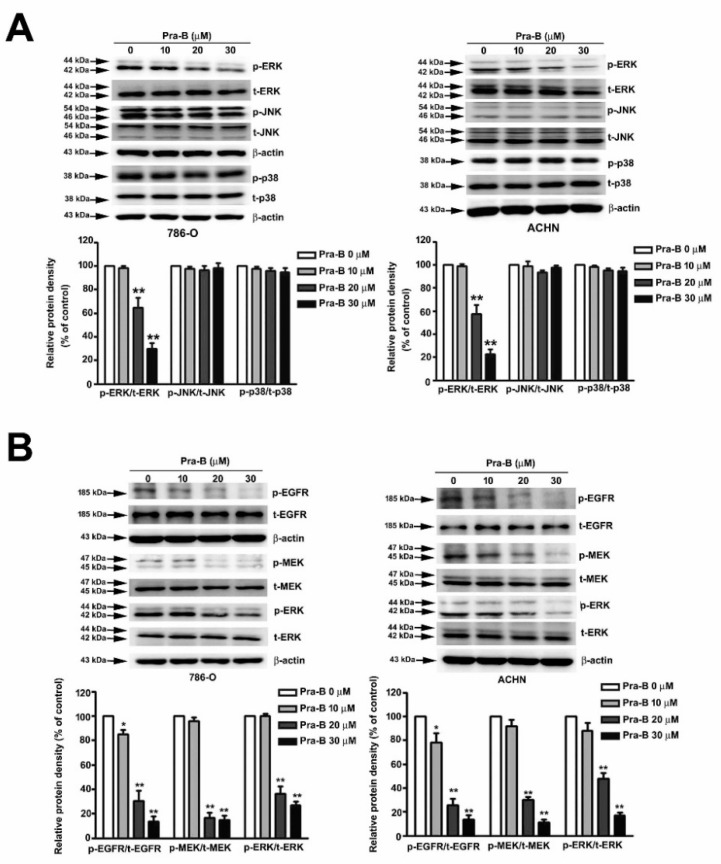
Pra-B suppressed epidermal growth factor receptor (EGFR)–MEK–ERK activation in 786-O and ACHN cells. (**A**) 786-O and ACHN cells were treated with various concentrations of Pra-B (0, 10, 20, and 30 μM) for 24 h, after which the cells were harvested to detect MAPKs-related proteins (p-ERK, t-ERK, p-JNK, t-JNK, p-p38, t-p38) and (**B**) the p-EGFR, t-EGFR, p-MEK, t-MEK, p-ERK, t-ERK protein expression levels through immunoblotting. The histogram represents the densitometric analysis of protein expression. β-actin was used as the loading control. * *p* < 0.05, ** *p* < 0.01 relative to the control. Data are presented in terms of mean ± SD, as determined in at least three independent experiments.

Numerous studies have explored the MAP kinase and its molecular mechanisms involved in the regulation of tumor cell migration and invasion [25]. To determine which MAPKs pathway was involved in downregulating of migration and invasive ability of Pra-B, we treated 786-O and ACHN cells with Pra-B (0, 10, 20, and 30 μM) and performed Western blot analysis to observe the activation of signaling pathways. As illustrated in Figure 4A, Pra-B inhibited the phosphorylation of ERK expression in 786-O and ACHN cells; however, the phosphorylation of JNK and p38 had no effects after Pra-B treatment.

### 2.5. Pra-B Attenuated EGF-Induced Migration and Invasion Ability through the Activation of EGFR–MEK–ERK Signaling Pathway

**Figure 5:**
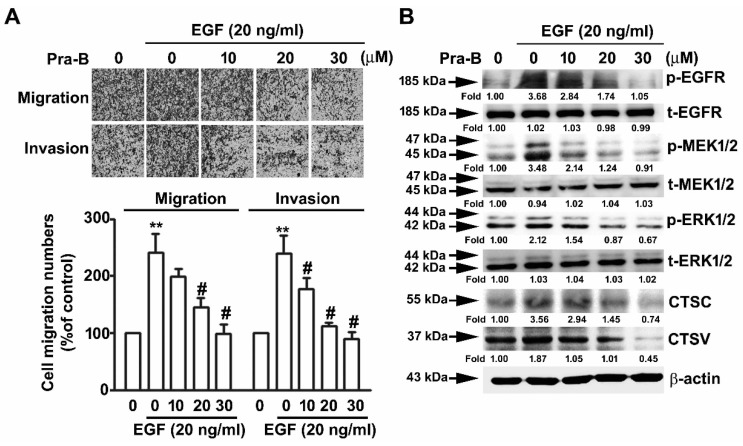
Pra-B attenuated epidermal growth factor-induced migration ability through the EGFR signaling pathway. The cells were pretreated with EGF (20 ng/mL) for 2 h and then incubated with various concentrations of Pra-B (0, 10, 20, and 30 μM) for 24 h. (**A**) Cell migration and invasion were measured using an in vitro migration and Matrigel-based invasion assay. Quantification of migrating cells presented in terms of percentage of control (0 μM) is shown as a histogram. (**B**) Cells were harvested to detect the p-EGFR, t-EGFR, p-MEK, t-MEK, p-ERK, t-ERK, CTSC, and CTSV protein expression levels through immunoblotting. β-actin was used as the loading control. The expression of these proteins was detected by densitometry as an average relative ratio compared to β-actin from three different experiments. ** *p* < 0.01 relative to the control. # *p* < 0.05 relative to EGF-treated alone. Data are presented in terms of mean ± SD, as determined in at least three independent experiments. Scale bar = 50 μm.

To examine the effect of Pra-B on the phosphorylation of EGFR–MEK–ERK pathways of RCC cells, we analyzed the expression of p-EGFR and p-MEK, which are upstream of p-ERK, and the results indicated that their phosphorylation expression was significantly inhibited upon Pra-B treatment 786-O and ACHN cells, not influencing total EGFR/MEK/ERK expression (Figure 4B). These results suggest that the EGFR–MEK–ERK activation signaling pathways are involved in the Pra-B-mediated inhibition of human RCC migration and invasion. To further determine the role of Pra-B in EGF-induced migration and invasion of 786-O cells, we found that Pra-B significantly inhibited EGF-induced cell migration of 786-O cells, compared with EGF-treated alone. Similar results were achieved with a Matrigel-based invasion assay (Figure 5A). In addition, to further clarify whether p-EGFR, p-MEK, p-ERK, CTSC, and CTSV in Pra-B-treated RCC cells were involved, we performed a Western blot analysis. As shown in Figure 5B, the increments of p-EGFR, p-MEK, p-ERK, CTSC, and CTSV expression through EGF induction were significantly decreased after Pra-B treatment at 20 and 30 μM. These findings indicate that the EGFR signaling pathway is involved in the inhibitory effect of Pra-B on EGF-induced EGFR–-MEK–ERK phosphorylation as well as CTSC and CTSV expression in 786-O cells.

## 3. Discussion

**Figure 6:**
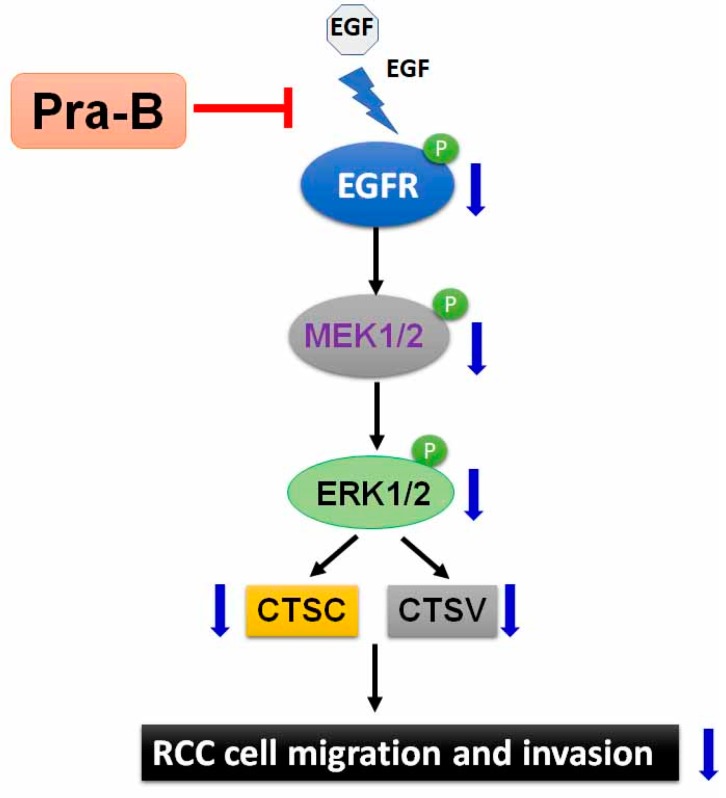
Illustration of how Pra-B inhibits the migration and invasion of human RCC cells through suppressing EGFR–MEK–ERK activation depending on CTSC and CTSV expression.

Several targeted drugs treating tumors by their histological and molecular subtype have been developed from natural products; these drugs have improved the treatment outcomes and distal metastasis of patients in the last decade [26,27]. Despite these notable advances, some unresolved issues remain, such as optimal drug selection for patients. Therefore, research on those bioactive agents that can be derived from natural products, especially their effectiveness for improving patient survival, is crucial. In this study, we demonstrated that a seselin-type coumarin, Pra-B, had no effect on cellular viability in normal human proximal tubule cells (HK2) and RCC cell lines (786-O and ACHN). However, Pra-B significantly inhibited RCC cells migration and invasion ability as well as downregulated CTSC and CTSV protein expression in a dose-dependent manner. Furthermore, Pra-B inhibited EGFR–MEK–ERK phosphorylation but had no effect on the JNK and p38 pathways. These results suggest that Pra-B can act as an antimetastatic agent through suppressing CTSC and CTSV expression as well as migration and invasion through downregulating the EGFR–MEK–ERK signaling cascade in RCC cells (Figure 6). In our previous study, we noted Pra-A’s antiproliferation and antimetastatic abilities in cervical cancer HeLa cells. In addition, we found that the effect of Pra-B was mediated by the PI3K/AKT/NF-κB signaling pathway in blocking cervical cancer cell metastasis [28]. Although Pra-B is a major bioactive compound of *Peucedanum praeruptorum* Dunn, its underlying mechanisms differ depending on the type of tumor cell it is interacting with.

Cancer cell metastasis is often accompanied by a series of ECM destruction leading to subsequent tumor cell migration and invasion [29]. In the context of carcinogenesis, cysteine cathepsins secreted into the extracellular environment contribute to tumor ECM degradation [30]. Recent studies have indicated that CTSD is involved in breast cancer invasion, and inducing CTSD may facilitate breast and gastric cancer cell migration [31,32]. In addition, studies have indicated that CTSB and CTSS mediate cancer progression and metastasis in lung cancer, renal cancer, and gastric cancer [33,34,35]. In our previous study, we discovered that CTSC is highly expressed in RCC cells and involved in inhibited the migratory and invasive ability in timosaponin AIII-treated RCC cells [24]. Our present results also demonstrate that the expression of CTSC and CTSV is inhibited by Pra-B as well as involved in the migration and invasion of RCC cells. Therefore, CTSC and CTSV may play crucial roles in the invasion of RCC cells and have a potential antimetastatic effect on RCC.

EGFR, also called ErbB1 or HER1, is a member of the ErbB family of receptors, which are transmembrane glycoproteins that stimulate their corresponding signaling pathway, including the KRAS–MEK1/2–ERK1/2 pathway, phosphoinositide 3-kinase (PI3K)–AKT kinase pathway, and STAT signaling pathway [36,37]. Evidence is accumulating for a critical role played by the EGFR-mediated signaling pathway in cell proliferation, survival, angiogenesis, and cancer metastasis [38]. A study observed the inhibition of CTSS-induced cancer-cell autophagy through the EGFR–MEK1/2–ERK1/2 cascade [39]. CTSD is highly expressed in colorectal tumors and positively correlated with the expression of EGFR [40]. Furthermore, the E3 ubiquitin ligase NEDD4 interacted with EGFR, which mediated lung cancer cell migration through activating CTSB [34]. The conclusions of the aforementioned studies suggest that the EGFR and CTS families closely regulate the carcinogenesis process. Numerous natural products have been identified as potential drugs based on their ability to target the EGFR signaling pathway. Butein, isolated from Rhus verniciflua, can inhibit EGFR phosphorylation, following the inhibition of the activation of its downstream signaling pathway [41]. An extract of Magnolia spp., Honokiol, has been demonstrated to be a phenolic compound with multiple activities, especially anticancer properties. Honokiol inhibited cancer cell migration and invasion through downregulating EGFR phosphorylation [42]. In conjunction with the aforementioned findings, our results demonstrated that through inhibiting the EGFR–MEK–ERK signaling pathway, CTSC and CTSV expression are involved in Pra-B’s suppression of EGF-induced migration and invasion in RCC cells.

Currently, tyrosine kinase inhibitors (TKIs) have been approved by the Food and Drug Administration for the treatment of RCC, and increasing evidence suggests that they are most effective and safe, with less toxicity [43]. It is well known that tyrosine kinase inhibitor (TKI)-targeted therapies such as sorafenib, pazopanib, or sunitinib that are combined with nature bioactive compounds or flavonoids may exert promoted antitumor or anti-invasive therapeutic effects and significantly decrease the systemic toxicity induced by combined targeted therapy; the results may be a possible lower dose [44]. However, we need to clarify two questions: First, what are the antitumor effects and molecular mechanisms of the combination of TKI-targeted therapy and Pra-B treatment of RCC cells? Second, are there any interactions or signaling pathways between the combination of TKI-targeted therapy and Pra-B? The answers to these questions should be studied in more focus and detail on the molecular mechanism of RCC in further research in the future.

## 4. Materials and Methods

### 4.1. Chemicals

Pra-B (purity > 98%) was purchased from ChemFace (Hubei, China). The origin stock solution of Pra-B is 100 mM in DMSO solvent. The final DMSO concentrations ranged from 0.01% for 10 μM Pra-B to 0.05% for 50 μM Pra-B. 3-(4,5-dimethylthiazol-2-yl)-2,5-diphenyl-tetrazolium bromide (MTT) was purchased from Sigma-Aldrich (St. Louis, MO, USA). Antibodies for CTSC, CTSV, t-ERK, p-JNK, t-JNK, p-p38, t-p38, p-MEK, t-MEK, p-EGFR, t-EGFR, and β-actin were purchased from Santa Cruz Biotechnology (Santa Cruz, CA, USA). The antibody for p-ERK was purchased from Cell Signaling Technology (Danvers, MA, USA).

### 4.2. Cell Lines and Cell Culture

The human renal carcinoma cell line 786-O (ATCC^®^ CRL-1932) was cultured in RPMI1640/high glucose medium. ACHN (ATCC^®^ CRL-1611) were maintained in minimum essential medium (MEM). All culture media was supplemented with 10% fetal bovine serum (FBS; Hyclone, MA, USA), 1% antibiotics (penicillin and streptomycin), 1 mM sodium pyruvate, and 100 mM nonessential amino acids. Human proximal tubular epithelial cells (HK-2) cultured in keratinocyte serum-free medium (KSFM; Thermo Fisher Scientific, MA, USA) were supplemented with epidermal growth factor (EGF; 10 ng/mL) and bovine pituitary extract (BPE; 40 mg/mL) and then incubated in a 5% CO_2_ humidified atmosphere at 37 °C.

### 4.3. Cell Viability

Cell viability was measured using MTT reagent. Cells were counted at 8 × 10^3^/100 μL and seeded in 96-well plates (Greiner Bio-one, Germany). After 24 h, the cells were cultured with 0.1% dimethyl sulfoxide (DMSO) or Pra-B (10, 20, 30, 40, and 50 μM) for 24 h. Subsequently, the medium was replaced with fresh medium containing MTT reagent (0.5 mg/mL) and incubated at 37 °C for 4 h. The product of formazan followed solubilization with 1 mL of isopropanol, and the color intensity was measured at 570 nm using a Multiskan MS ELISA reader (Labsystems, Helsinki, Finland).

### 4.4. Cell Migration and Invasion

Migration and invasion assays were performed per a previously described method [45]. In brief, cells were counted at 4 × 10^5^/3 mL and seeded in a 6-cm dish (Greiner Bio-one). After 24 h, the cells were treated first with Pra-B (10, 20, and 30 μM) for 24 h, and subsequently using 48-well modified Boyden chambers containing polycarbonate filter inserts (Millipore) with 8-μm pores in RPMI1640 or MEM medium. These filter inserts were coated with Matrigel (10 μL, BD Biosciences) prior to the invasion assay. Cells were counted at 2 × 10^4^/50 μL and placed in the upper part of the chamber, which contained serum-free RPMI1640 or MEM medium, before being incubated for 16 h. The migrated cells were counted using an inverted microscope (200×, Leica). Three sets of five microscopic fields were counted for each sample.

### 4.5. Quantitative RT-PCR (qRT-PCR)

The qRT-PCR was performed per a previously described method [46]. In brief, total RNA was isolated from cells using Trizol Reagent (Invitrogen, USA) and quantified. Total RNA quantity was estimated using a U-2900 Spectrophotometer (HITACHI, Fukuoka, Japan). cDNA was synthesized from 1 μg of RNA using a GoScript™ Reverse Transcription System (Promega, Madison, WI, USA) according to the manufacturer’s instructions. Real-time PCR was performed using GoTaq qPCR Master Mix reagents (Promega, Madison, WI, USA) in an ABI PRISM 7700 real-time PCR system (Applied Biosystems). RT-qPCR primer was shown as CTSA, forward: 5′-GTCGCCCAGAGCAATTTTGAG-3′; reverse: 5′-TCTCCCCGGTCAGGAAAAGTT-3′. CTSB, forward: 5′-GAGCTGGTCAACTATGTCAACA-3′; reverse: 5′-GCTCATGTCCACGTTGTAGAAGT-3′. CTSC, forward: 5′-CCAACTGCACCTATCTTGACC-3′; reverse: 5′-AAGGCAAACCACTTGTAGTCATT-3′. CTSS, forward: 5′-GCCTGATTCTGTGGACTGG-3′; reverse: 5′-GATGTACTGGAAAGCCGTTGT-3′. CTSV, forward: 5′-CGTGACGCCAGTGAAGAATCA-3′; reverse: 5′-CGCTCAGTGAGACAAGTTTCC-3′. CTSX, forward: 5′-CAGCGGATCTGCCCAAGAG-3′; reverse: 5′-CGATGACGTTCTGCACGGA-3′. GAPDH forward: 5′-CATCATCCCTGCCTCTACTG-3′; reverse: 5′-GCCTGCTTCACCACCTTC-3′. GAPDH was used as an internal control, and the difference in threshold cycle (Ct) between treated and untreated cells was determined using the 2^−ΔΔ*C*t^ method.

### 4.6. Western Blotting

Western blotting was performed per a previously described method [47]. Cells were washed with cold PBS and resuspended in lysis buffer (20 mM Tris; pH 8.0, 1 mM EDTA; pH 8.0, 150 mM NaCl, and 0.5% NP-40) plus a cocktail of proteinase and phosphatase inhibitors (Roche Molecular Biochemicals). After sonication for 10 s and incubation for 20 min on ice, the supernatant was collected by centrifugation at 13,000× *g* for 15 min at 4 °C. Equal amounts of proteins (20 µg) of the sample were separated using 10–12% SDS-PAGE and transferred onto polyvinylidene fluoride membrane (Life Technologies, CA, USA). Membranes were blocked with blocking buffer (5% nonfat dry milk; 20 mM Tris-HCl, pH 7.6; 150 mM NaCl; and 0.1% Tween 20) for 1 h at room temperature. Membranes were incubated with antibody for CTSC (1:1000), CTSV (1:1000), p-ERK (1:1000), t-ERK (1:1000), p-JNK (1:1000), t-JNK (1:1000), p-p38 (1:1000), t-p38 (1:1000), p-MEK (1:1000), t-MEK (1:1000), p-EGFR (1:1000), t-EGFR (1:2000), and β-actin (1:2000) in blocking buffer on an orbit shaker at 4 °C overnight, followed by incubation with horseradish peroxidase–linked secondary antibodies (1:10000) for 2 h at 4 °C. The proteins were visualized using ECL reagents (EMD Millipore, USA) under an LAS-4000 mini luminescent image analyzer.

### 4.7. Statistical Analysis

Differences in experimental results were statistically analyzed using one-way analysis of variance in SPSS (version 10.0). Differences between the control or Pra-B–treated groups were analyzed by one-way ANOVA and Dunnett’s post hoc test. Results were expressed in terms of mean ± standard deviation in triplicate, with a *p*-value < 0.05 or < 0.01 indicating statistical significance.

## 5. Conclusions

The present results suggest that the EGFR–MEK–ERK pathways may be key mediators of Pra-B’s antimetastatic action through the inhibition of the expression of CTSC and CTSV. Ours is the first study to demonstrate the roles and possible mechanisms of EGFR, CTSC, and CTSV on the metastasis of human RCC. Our findings uncover the molecular mechanisms underlying the role of Pra-B, and CTSC/CTSV is considered a potential antimetastatic target against RCC cells.
